# A Non-randomized Clinical Trial on the Effectiveness and Complications of Two Different Streptokinase Dosages in Elderly Patients With ST-Elevation Myocardial Infarction (STEMI)

**DOI:** 10.7759/cureus.107710

**Published:** 2026-04-25

**Authors:** Subhash Chandra, Prachi Jain Rai, Nouratan Singh, Aaditya Shivhare

**Affiliations:** 1 Cardiology, Uttar Pradesh University of Medical Sciences, Etawah, IND; 2 Transfusion Medicine, Uttar Pradesh University of Medical Sciences, Etawah, IND

**Keywords:** complication, mortality, older, st elevation myocardial infarction, streptokinase

## Abstract

Background: Streptokinase is widely used for thrombolysis in acute myocardial infarction (AMI), but concerns about complications have led to its underutilization in elderly patients. This study aimed to compare the efficacy and safety of standard-dose, i.e., 15 lakh international units (IU), versus half-dose (7.5 lakh IU) streptokinase in elderly patients with ST-elevation myocardial infarction (STEMI).

Materials and methods: This prospective, observational study included 103 patients with STEMI aged over 70 years, admitted within 12 hours of symptom onset. Patients were divided into two groups receiving either 15 lakh IU (n = 59) or 7.5 lakh IU (n = 44) of streptokinase. Efficacy was assessed by ST-segment resolution ≥ 50% after 90 minutes. Complications and mortality were monitored in both groups.

Results: Baseline demographic and clinical characteristics were comparable between the groups. Improvement in left ventricular ejection fraction (LVEF) from admission to one month was similar (2.75% vs. 2.84%, p = 0.885), and rates of reperfusion success (78.0% vs. 77.3%) and ST-segment resolution ≥ 50% (61.0% vs. 59.1%) did not differ significantly. Complications were more frequent in the 7.5 lakh IU group, particularly major bleeding (11.4% vs. 3.4%), though differences were not statistically significant. In-hospital mortality was low and comparable between the two groups (5.1% vs. 6.8%, p = 0.690).

Conclusion: Half-dose streptokinase demonstrated comparable efficacy to the standard dose in achieving myocardial reperfusion in elderly patients having STEMI.

## Introduction

Acute myocardial infarction (AMI) remains a leading cause of mortality among the elderly population, particularly those over 65 years of age. In India and other South Asian countries, cardiovascular diseases account for a substantial proportion of mortality in older adults, with AMI contributing significantly; the prevalence of self-reported cardiovascular disease among adults aged ≥45 years is approximately 30%, rising to 38% in those ≥70 years, and ischemic heart disease prevalence has increased markedly in both rural and urban settings over recent decades [1]. In India, where 6.8% of the population is over 60 years old, AMI accounts for 80% of deaths in this age group. The mortality rate for elderly patients post-AMI is twice that of younger patients [2], underscoring the critical need for effective treatment strategies tailored to this vulnerable demographic.

Thrombolytic therapy has been well-established as a crucial strategy in the management of ST-elevation myocardial infarction (STEMI). As demonstrated in one of the meta-analyses, timely administration of fibrinolytic agents can significantly reduce mortality [3]. Also, literature showed that streptokinase (SK) prevents approximately 30 early deaths per 1,000 patients when administered within six hours of symptom onset [4]. Among the various fibrinolytic agents available, SK remains the most widely used, particularly in economically constrained healthcare systems, due to its lower cost compared to newer-generation thrombolytics [5]. However, SK administration is not without risks, especially in older patients who experience higher rates of complications and mortality associated with its use. This has led to a growing consensus that dosage alterations or additional precautions may be necessary when administering SK to elderly patients. While previous observational studies (e.g., Kumar et al.) and randomized trials of other agents have explored dose reduction, this study uniquely contributes real-world, prospective observational data on half-dose versus standard-dose SK specifically in Indian elderly patients (>70 years) with STEMI in a resource-limited tertiary care setting, focusing on both electrocardiogram (ECG)/biochemical reperfusion markers and complication profiles where evidence remains limited [2].

Considering these findings and the imperative to mitigate complications in elderly patients with STEMI, our study aims to evaluate the efficacy and safety of a reduced dose of SK 7.5 lakh international units (IU) compared to the conventional dose (15 lakh IU) in achieving myocardial reperfusion in elderly patients with STEMI. Additionally, we seek to assess whether this lower dose regimen results in any significant changes in the incidence of complications. Elderly patients often have lower body mass, reduced thrombus burden, and altered fibrinolytic responses; pharmacodynamic studies suggest that lower SK doses may achieve sufficient localized plasminogen activation with reduced systemic fibrinogenolysis, potentially preserving efficacy while mitigating bleeding risk.

Objectives of the study

This prospective observational study aimed to compare the efficacy and safety of standard-dose (15 lakh IU) versus half-dose (7.5 lakh IU) SK in patients > 70 years with STEMI. The primary endpoint was the proportion of patients achieving ST-segment resolution ≥ 50% on ECG at 90 minutes after SK administration. Secondary endpoints included reperfusion success (complete or partial), improvement in left ventricular ejection fraction (LVEF) from admission to one month, in-hospital complications (including major bleeding), and in-hospital mortality. As an observational study, no formal superiority, equivalence, or non-inferiority hypothesis was predefined; the analysis was exploratory, with the intent to generate hypotheses regarding dose-related differences in real-world clinical practice.

## Materials and methods

This was a prospective, observational study conducted at a tertiary care center between January 2025 and September 2025. The study was approved by the Institutional Ethics Committee (207/2024-25). A patient consent form was collected prior to enrolling the patients.

Study design and population

**Figure 1:**
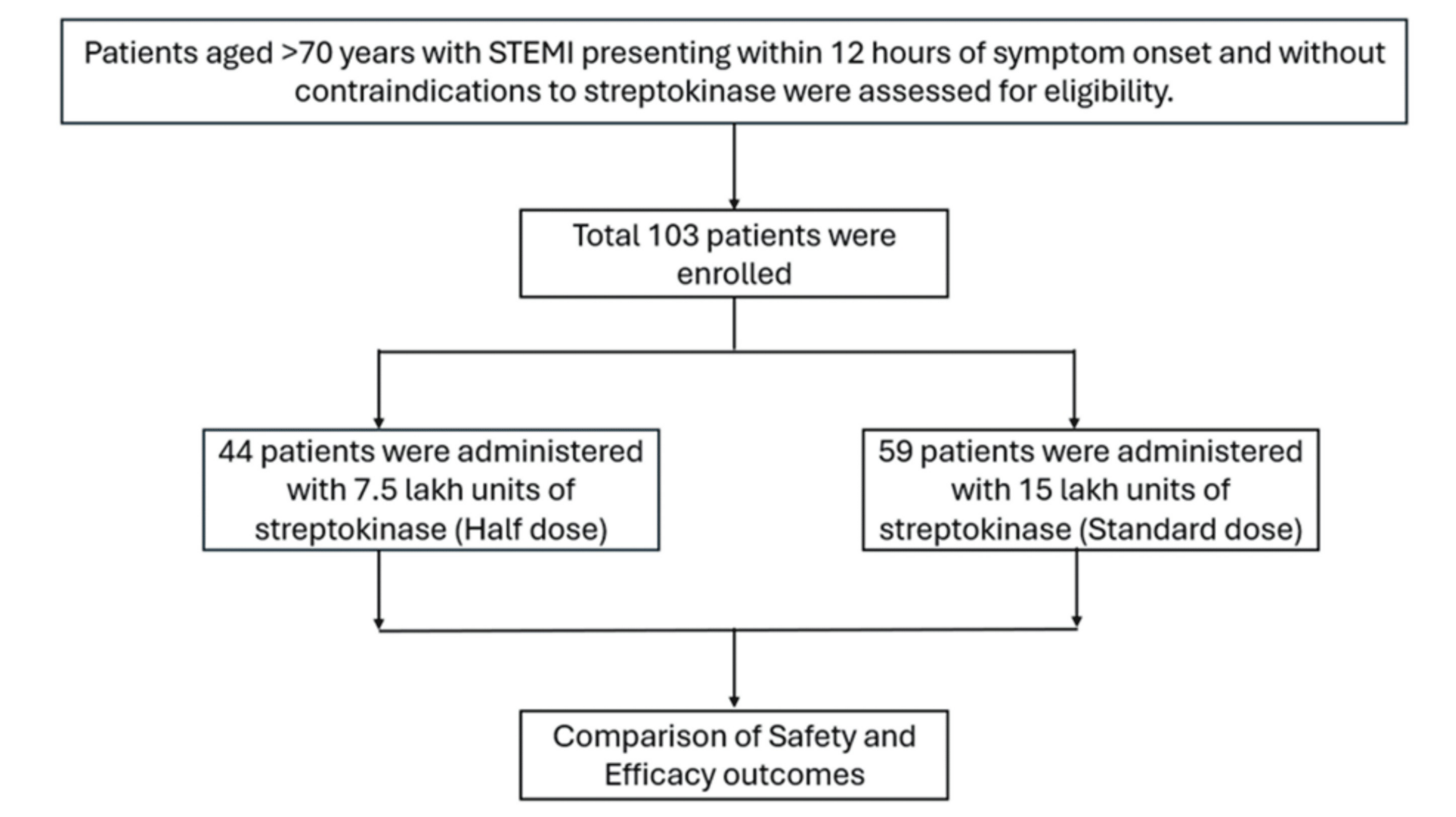
Flow chart for study population selection STEMI: ST-elevation myocardial infarction

The study included 103 patients aged over 70 years with STEMI who were admitted to the cardiology department. Eligibility criteria required patients to present within 12 hours of symptom onset and have no contraindications to SK thrombolysis. Those with absolute or relative contraindications to SK were excluded. The eligible patients were divided into two groups: one group received an intravenous dose of 7.5 lakh units of SK, while the other group was administered the standard dose of 15 lakh units of SK (Figure 1).

Data collection

**Figure 2:**
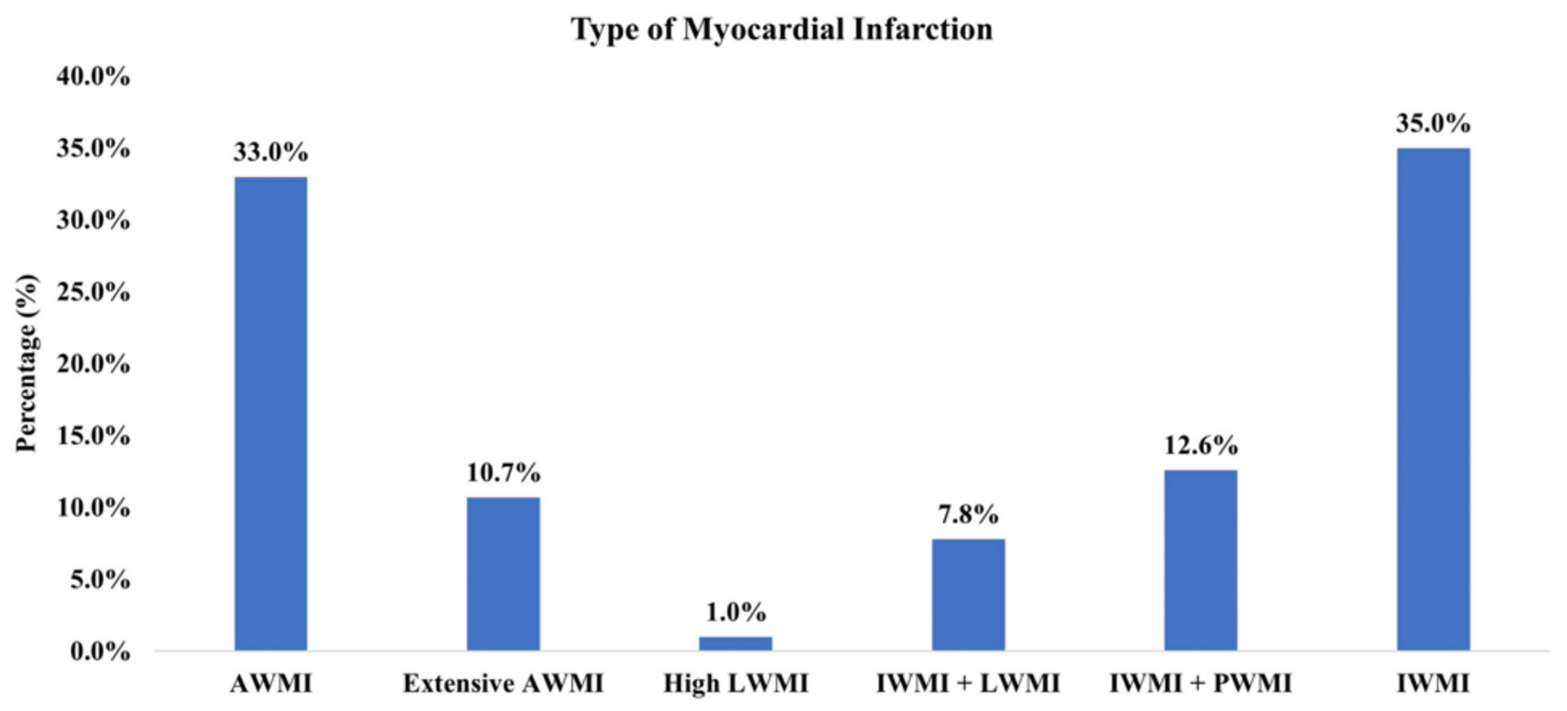
Types of myocardial infarction in the study patients AWMI: anterior wall myocardial infarction; IWMI: inferior wall myocardial infarction; LWMI: lateral wall myocardial infarction; PWMI: posterior wall myocardial infarction

Clinical and biochemical profiles were obtained for each patient, including a detailed medical history. The STEMI diagnosis was based on the criteria established according to the Universal Definition of Myocardial Infarction (Figure 2) [6]. Following SK administration, patients were monitored after 90 minutes to evaluate ST-segment resolution (50% or more) on ECG. The Killip classification was employed to assess mortality risk [7]. Both groups were observed for complications following SK administration. This standardized approach allowed for a thorough comparison of outcomes between the two dosage groups. All patients received standard guideline-directed therapy, including dual antiplatelet therapy (aspirin 162-325 mg loading dose followed by 75 mg daily + clopidogrel 300 mg loading dose followed by 75 mg daily), and anticoagulation with unfractionated heparin or low-molecular-weight heparin as per institutional protocol.

Efficacy and safety endpoints

LVEF was measured by two-dimensional echocardiography at admission (within 24 hours of admission) and at the one-month follow-up using Simpson’s biplane method. Echocardiograms were performed and interpreted by cardiologists who were not blinded to treatment allocation, as this was an observational study. Efficacy of thrombolysis was evaluated using several parameters. The primary endpoint was ST-segment resolution of 50% or more on ECG at 90 minutes after SK administration. Secondary endpoints included reperfusion success, categorized as complete or partial, and improvement in LVEF from admission to one month, measured by two-dimensional echocardiography. Biochemical markers, including peak levels of creatine phosphokinase-myocardial band (CPK-MB) and Troponin I, were monitored to assess myocardial reperfusion, and the time to peak enzyme levels within 0-12 hours vs. 12-24 hours was recorded as supportive data. Safety outcomes were systematically documented, including the incidence of major and minor bleeding, hypotension, anaphylaxis, post-myocardial infarction (MI) angina, and renal dysfunction. All patients were closely monitored during hospitalization, and any complications related to SK therapy were recorded according to standardized definitions. In-hospital mortality was also tracked as a critical safety endpoint.

Statistical analysis

Statistical analysis was conducted using R software version 4.3.3 (The R Foundation, Vienna, Austria). Continuous variables were expressed as mean ± standard deviation, and categorical variables were presented as numbers and percentages. Normality of data was assessed using the Shapiro-Wilk test. For normally distributed data, comparisons between groups were performed using Student's t-test, while non-normally distributed data were analyzed using the Mann-Whitney U test. Categorical variables were compared using the chi-squared test or Fisher's exact test, as appropriate. All reported p-values were two-sided, with values less than 0.05 considered statistically significant. All analyses were performed on an intention-to-treat basis, including all 103 enrolled patients. The primary outcome was defined as ST-segment resolution ≥ 50% at 90 minutes. All other endpoints (reperfusion success, LVEF improvement, complications, and mortality) were secondary. No formal adjustment for multiplicity was applied, given the exploratory/pilot nature of the study.

## Results

In this study, a total of 103 patients were enrolled; 59 (57.3%) received SK 15 lakh IU and 44 (42.7%) received SK 7.5 lakh IU. Baseline characteristics were broadly similar between the two groups (Table 1). Efficacy parameters are detailed in Table 1. ST-segment resolution ≥ 50% was achieved in n = 36 (61.0%) of patients in the 15 lakh IU group and n = 26 (59.1%) in the 7.5 lakh IU group (p = 0.843). Reperfusion success was almost identical (n = 46 (78.0%) vs. n = 34 (77.3%), p = 0.933). Improvement in mean LVEF from admission to one month was also comparable (2.75% ± 3.15% vs. 2.84% ± 3.10%, p = 0.885). Mean CPK-MB levels were numerically higher in the 15 lakh IU group (105.5 ± 51.6 vs. 94.1 ± 58.6, p = 0.299). In-hospital mortality was observed in three patients in each group (n = 3 (5.1%) vs. n = 3 (6.8%), p = 0.690).

**Table 1:** Efficacy parameters for streptokinase doses Data are represented as n (%). p-value < 0.05 was considered statistically significant. CPK-MB: creatine phosphokinase-myocardial band; IU: international unit; LVEF: left ventricular ejection fraction; SD: standard deviation

Efficacy parameter	Total (N = 103 patients)	Streptokinase 15 lakh IU (N = 59 patients)	Streptokinase 7.5 lakh IU (N = 44 patients)	p-value
ST-segment resolution ≥ 50%, n (%)	62 (60.2)	36 (61.0)	26 (59.1)	0.843
Reperfusion success (complete/partial), n (%)	80 (77.7)	46 (78.0)	34 (77.3)	0.933
LVEF improvement (admission to 1 month), mean ± SD	2.78 ± 3.12	2.75 ± 3.15	2.84 ± 3.10	0.885
Peak Troponin I time 0-12 hours, n (%)	55 (53.4)	31 (52.5)	24 (54.5)	0.840
CPK-MB level, mean ± SD	100.61 ± 54.74	105.47 ± 51.62	94.09 ± 58.64	0.299
In-hospital mortality, n (%)	6 (5.8)	3 (5.1)	3 (6.8)	0.690

Complications related to SK therapy are summarized in Table 2. Overall complication rates were numerically higher in the 7.5 lakh IU group (n = 9 (20.5%) vs. n = 4 (6.8%), p = 0.491). Major bleeding occurred in n = 5 (11.4%) of patients in the 7.5 lakh IU group compared with n = 2 (3.4%) in the 15 lakh IU group (p = 0.134).

**Table 2:** Complications due to streptokinase among patients with different doses Data are represented as n (%). p-value < 0.05 was considered statistically significant. IU: international unit

Complications	Total (N = 103 patients)	Streptokinase 15 lakh IU (N = 59 patients)	Streptokinase 7.5 lakh IU (N = 44 patients)	p-value
Complications of streptokinase	13 (12.6)	4 (6.8)	9 (20.5)	0.491
Major bleeding	7 (6.8)	2 (3.4)	5 (11.4)	0.134
Minor bleeding	1 (1.0)	1 (1.7)	0 (0.0)	>0.999
Anaphylaxis	1 (1.0)	0 (0.0)	1 (2.3)	0.427
Hypotension	4 (3.9)	1 (1.7)	3 (6.8)	0.310

The proportion of males was slightly higher in the 15 lakh IU group (n = 44 (74.6%) vs. n = 30 (68.2%), p = 0.475) in Table 3. The prevalence of smoking (n = 43 (72.9%) vs. n = 29 (65.9%), p = 0.445) and hypertension (n = 18 (30.5%) vs. n = 15 (34.1%), p = 0.700) was comparable.

**Table 3:** Comparison of baseline and clinical demographics between the streptokinase 15 lakh IU and 7.5 lakh IU groups Data are represented as n (%). p-value < 0.05 was considered statistically significant. H/O CAD: history of coronary artery disease; IU: international unit

Variables	Total (N = 103 patients)	Streptokinase 15 lakh IU (N = 59 patients)	Streptokinase 7.5 lakh IU (N = 44 patients)	p-value
Gender				
Male	74 (71.8)	44 (74.6)	30 (68.2)	0.475
Female	29 (28.2)	15 (25.4)	14 (31.8)	
Risk factors				
Smoking	72 (69.9)	43 (72.9)	29 (65.9)	0.445
Hypertension	33 (32.0)	18 (30.5)	15 (34.1)	0.700
Diabetes mellitus	33 (32.0)	15 (25.4)	18 (40.9)	0.096
Family H/O CAD	17 (16.5)	11 (18.6)	6 (13.6)	0.498
Dyslipidemia	15 (14.6)	7 (11.9)	8 (18.2)	0.369

Laboratory characteristics including LVEF, serum creatinine, peak CPK-MB, and Killip classification at presentation are described in Table 4. Baseline mean LVEF was nearly identical in both groups (39.08% ± 7.73% vs. 39.07% ± 8.46%, p = 0.992). At one month, mean LVEF improved to 41.83% ± 7.42% vs. 41.9% ± 7.40% in both groups, with no significant difference (p = 0.958). Mean serum creatinine levels were similar (1.14 ± 0.30 vs. 1.16 ± 0.32 mg/dL, p = 0.806). Killip class distribution showed that more patients in the 7.5 lakh IU group were in Class I (n = 29 (65.9%) vs. n = 30 (50.8%)), while higher Killip classes were slightly more frequent in the 15 lakh IU group, though these differences were not significant (p = 0.299).

**Table 4:** Comparison of variables between the streptokinase 15 lakh IU and 7.5 lakh IU groups Data are represented as n (%) and mean ± SD. p-value < 0.05 was considered statistically significant. CPK-MB: creatine phosphokinase-myocardial band; IU: international unit; LVEF: left ventricular ejection fraction; SD: standard deviation

Variables	Total (N = 103 patients)	Streptokinase 15 lakh IU (N = 59 patients)	Streptokinase 7.5 lakh IU (N = 44 patients)	p-value
LVEF, % (admission)	39.08 ± 8.01	39.08 ± 7.73	39.07 ± 8.46	0.992
LVEF, % (1 month)	41.86 ± 7.38	41.83 ± 7.42	41.91 ± 7.40	0.958
Serum creatinine	1.15 ± 0.31	1.14 ± 0.30	1.16 ± 0.32	0.806
Time from streptokinase to peak CPK-MB				
0-12 hours	55 (53.4)	31 (52.5)	24 (54.5)	0.840
12-24 hours	48 (46.6)	28 (47.5)	20 (45.5)	
Killip classification				
Class 1	59 (57.3)	30 (50.8)	29 (65.9)	0.299
Class 2	31 (30.1)	19 (32.2)	12 (27.3)	
Class 3	11 (10.7)	8 (13.6)	3 (6.8)	
Class 4	2 (1.9)	2 (3.4)	0 (0.0)	

As presented in Table 5, anthropometric measures and hemodynamic parameters were comparable between groups. Renal dysfunction occurred in n = 10 (16.9%) of the 15 lakh IU group and n = 7 (15.9%) of the 7.5 lakh IU group (p = 0.888). Triple-vessel disease was more frequent in the lower-dose group (n = 6 (13.6%) vs. n = 4 (6.8%), p = 0.319), whereas post-MI angina occurred at similar rates (n = 9 (20.5%) vs. n = 9 (15.3%), p = 0.492). Although no statistically significant differences were observed in efficacy or safety endpoints, the study was underpowered to detect clinically meaningful differences, especially for less frequent events such as major bleeding.

**Table 5:** Comparison of variables between the streptokinase 15 lakh IU and 7.5 lakh IU groups Data are represented as n (%). p-value < 0.05 was considered statistically significant. bpm: beats per minute; IU: international unit; MI: myocardial infarction

Patient’s characteristics	Total (N = 103 patients)	Streptokinase 15 lakh IU (N = 59 patients)	Streptokinase 7.5 lakh IU (N = 44 patients)	p-value
Height	162.03 ± 7.46	162.27 ± 7.65	161.70 ± 7.27	0.705
Weight	62.50 ± 7.87	62.42 ± 8.27	62.61 ± 7.38	0.904
Pulse bpm	88.56 ± 23.09	87.44 ± 23.29	90.07 ± 22.99	0.570
Systolic pressure	114.64 ± 25.02	113.68 ± 25.38	115.93 ± 24.77	0.653
Diastolic pressure	71.49 ± 15.27	71.80 ± 15.64	71.07 ± 14.93	0.812
Renal dysfunction	17 (16.5)	10 (16.9)	7 (15.9)	0.888
Triple vessel disease	10 (9.7)	4 (6.8)	6 (13.6)	0.319
Post-MI angina	18 (17.5)	9 (15.3)	9 (20.5)	0.492

## Discussion

The present study evaluated the efficacy and safety of a reduced dose of SK (7.5 lakh IU) compared to the conventional dose (15 lakh IU) in elderly patients with acute STEMI. The major findings of our study were the following: (1) reperfusion rates, assessed by ST-segment resolution ≥ 50% and combined complete/partial reperfusion, were comparable between the two groups; (2) LVEF improvement at one month was similar in both groups; (3) the incidence of complications, including major bleeding, hypotension, and anaphylaxis, did not differ significantly between groups; and (4) in-hospital mortality was also comparable (5.1% in conventional dose vs. 6.8% in half dose).

In our study, ST-segment resolution ≥ 50% was achieved in n = 36 (61.0%) of patients in the 15 lakh IU group and n = 26 (59.1%) in the 7.5 lakh IU group (p = 0.843), while overall reperfusion success (complete or partial) was n = 46 (78.0%) vs. n = 34 (77.3%) (p = 0.933). These findings suggest that the lower half dose of SK has no statistically significant difference compared to the conventional dose in achieving myocardial reperfusion in elderly STEMI patients. Our results align with previous studies, including Ahmad and Ishaq [8], which demonstrated that half-dose SK produces comparable reperfusion to the full dose while potentially reducing adverse events in older populations. The time to peak Troponin I and CPK-MB within 12 hours was also similar between groups, further supporting that no statistically significant difference was observed in the reduced dose. Mechanistically, SK facilitates thrombolysis through plasminogen activation. Previous pharmacodynamic studies have shown that lower doses may still achieve sufficient local thrombolysis in elderly patients, who often have smaller thrombus burden and lower body mass, minimizing systemic exposure without compromising efficacy [9].

In our study, major bleeding occurred in n = 2 (3.4%) of patients receiving 15 lakh IU and n = 5 (11.4%) of those receiving 7.5 lakh IU (p = 0.134), while hypotension occurred in n = 1 (1.7%) and n = 3 (6.8%) of patients, respectively (p = 0.310). Although numerically higher in the reduced-dose group, these differences were not statistically significant, and no cases of anaphylaxis or intracranial hemorrhage were reported. These results are consistent with prior literature suggesting that half-dose SK maintains an acceptable safety profile, particularly in elderly patients who are at higher baseline risk for bleeding [10,11]. The slightly higher incidence of hypotension in the reduced-dose group may be explained by the small sample size. SK-induced hypotension is typically transient and responds well to intravenous saline infusion without additional intervention [12-14]. In-hospital mortality in our study was low in both groups (n = 3 (5.1%) in the 15 lakh IU group and n = 3 (6.8%) in the 7.5 lakh IU group, p = 0.690), which is lower than that reported in the ASK-ROMANIA registry (accelerated SK 7.38%, standard SK 11.6%) [15]. Among patients receiving late treatment, i.e., more than six hours after symptom onset, our results were comparable with ASK-ROMANIA, suggesting that reduced-dose SK remains effective even in delayed presentations. These findings support the favorable safety profile of half-dose SK in elderly STEMI patients, without compromising short-term survival.

Our findings are comparable to findings in a study by Siriwattana et al. [16], where lower-dose SK achieved similar reperfusion and mortality rates to standard dosing, with no increase in major complications. Similarly, a study by Kumar et al. [2] demonstrated that half-dose SK produced comparable rates of ECG and biochemical evidence of reperfusion, with a trend toward fewer adverse events. These consistencies reinforce the clinical viability of reduced-dose thrombolysis in elderly STEMI populations.

Improvement in LVEF from admission to one month was similar between groups (mean increase ~2.8%), indicating preservation of left ventricular function irrespective of SK dose, suggesting that reduced-dose therapy does not compromise short-term survival. The similarity in baseline demographics, Killip class distribution, and risk factors between the two groups strengthens the validity of our findings.

This study has several important limitations. First, the non-randomized observational design introduces potential selection bias and confounding by indication, as dose allocation was at the treating cardiologist’s discretion. Second, the modest sample size (N = 103) resulted in limited statistical power, particularly for detecting differences in safety outcomes such as major bleeding. Third, no multivariable adjustment was performed for potential confounders (e.g., Killip class, time to treatment, and comorbidities). Fourth, echocardiographic assessors were not blinded to treatment allocation. Fifth, follow-up was limited to one month, with no long-term outcome data. Finally, as a single-center study conducted in India, generalizability to other populations or settings may be limited. Elderly patients frequently have lower body mass and potentially smaller thrombus burden; however, whether this fully explains similar reperfusion with half-dose SK remains uncertain and requires confirmation in dedicated pharmacodynamic studies. Further, a large-scale study is needed to confirm these results and explore potential variations in outcomes across different age groups.

Clinical relevance

The observed mean LVEF improvement of approximately 2.8% in both groups is modest and of uncertain clinical significance for long-term outcomes. Similarly, ST-segment resolution ≥ 50% occurred in ~60% of patients, a rate consistent with historical thrombolysis data but lower than contemporary primary percutaneous coronary intervention (PCI) benchmarks. These metrics suggest preserved short-term ventricular function but highlight that neither dose achieved optimal reperfusion in a substantial proportion of elderly patients.

## Conclusions

In this prospective observational study of elderly patients (>70 years) presenting with STEMI, a reduced half-dose SK regimen (7.5 lakh IU) appeared to offer a promising alternative to the standard full dose (15 lakh IU). The approach achieved comparable myocardial reperfusion while maintaining a similar safety profile, including in-hospital mortality. A numerical trend toward higher complications, particularly major bleeding (11.4% vs. 3.4%), was observed in the half-dose group, although not statistically significant. Given the non-randomized design and small sample size, these findings do not support claims of safety equivalence between the two dosing regimens. These results are hypothesis-generating only and should not be interpreted as practice-changing. The non-randomized observational design and limited statistical power preclude firm conclusions regarding comparative efficacy or safety. Larger, multicenter randomized controlled trials with adequate sample size, predefined non-inferiority margins for both efficacy and safety endpoints, and proper adjustment for confounders are required before any reduced-dose strategy can be considered for routine clinical use in elderly patients with STEMI. These findings should therefore be considered hypothesis-generating rather than definitive evidence for clinical practice change.
